# InterAcT: A generic keypoints-based lightweight transformer model for recognition of human solo actions and interactions in aerial videos

**DOI:** 10.1371/journal.pone.0323314

**Published:** 2025-05-14

**Authors:** Mubashir Shah, Tahir Nawaz, Rab Nawaz, Nasir Rashid, Muhammad Osama Ali

**Affiliations:** 1 Department of Mechatronics Engineering, College of Electrical and Mechanical Engineering, National University of Sciences and Technology, Islamabad, Pakistan; 2 Deep Learning Lab, School of Interdisciplinary Engineering and Science, National University of Sciences and Technology, Islamabad, Pakistan; 3 Faculty of Computing, Capital University of Science and Technology, Islamabad, Pakistan; Government College University Faisalabad, PAKISTAN

## Abstract

Human action recognition forms an important part of several aerial security and surveillance applications. Indeed, numerous efforts have been made to solve the problem in an effective and efficient manner. Existing methods, however, are generally aimed to recognize either solo actions or interactions, thus restricting their use to specific scenarios. Additionally, the need remains to devise lightweight and computationally efficient models to make them deployable in real-world applications. To this end, this paper presents a generic lightweight and computationally efficient Transformer network-based model, referred to as InterAcT, that relies on extracted bodily keypoints using YOLO v8 to recognize human solo actions as well as interactions in aerial videos. It features a lightweight architecture with 0.0709M parameters and 0.0389G flops, distinguishing it from the AcT models. An extensive performance evaluation has been performed on two publicly available aerial datasets: Drone Action and UT-Interaction, comprising a total of 18 classes including both solo actions and interactions. The model is optimized and trained on 80% train set, 10% validation set and its performance is evaluated on 10% test set achieving highly encouraging performance on multiple benchmarks, outperforming several state-of-the-art methods. Our model, with an accuracy of 0.9923 outperforms the AcT models (micro: 0.9353, small: 0.9893, base: 0.9907, and large: 0.9558), 2P-GCN (0.9337), LSTM (0.9774), 3D-ResNet (0.9921), and 3D CNN (0.9920). It has the strength to recognize a large number of solo actions and two-person interaction classes both in aerial videos and footage from ground-level cameras (grayscale and RGB).

## Introduction

Human action recognition involves automatically analyzing and comprehending varying actions in videos, which form a part of several security and surveillance applications [1,2]. Broadly, existing action recognition approaches may be categorized into non-vision-based and vision-based methods.

*Non-vision-based methods* rely on data from wearable sensors [3] or non-wearable sensors [4]. Wearable sensors refer to body-worn devices/accessories to capture physiological signals or motion data and include, for example, accelerometers, magnetometers, gyroscopes, smart watches, etc. Non-wearable sensors are stationary or mobile devices that generally capture environmental data without direct contact with the body and include, for example, sound sensors, pressure sensors, temperature sensors etc. Examples of different action recognition approaches employed in non-vision-based methods include Deep Neural Networks (DNN) [5], Convolutional Neural Networks (CNN) [6], Autoencoders [7], Restricted Boltzmann Machine (RBM) [8], and Recurrent Neural Network (RNN) [9] and Hybrid models [10]. While these methods are generally computationally less costly and robust to deal with varying illumination, they have limitations in terms of scalability, accuracy and generalizability.

*Vision-based methods*, on the other hand, use image or video data coming from cameras to perform action recognition with better performance and generalizability [11]. Traditional vision-based methods comprise of two key components [12]: action representation and action classification. Action representation is the process of converting video or image data into feature vectors [13], whereas action classification then infers the class labels using the encoded data in feature vectors [14]. Indeed, the traditional machine learning methods [15] rely on hand-crafted feature extraction methods such as Histogram of Oriented Gradients (HOG) [16], Optical Flow [17], Spatiotemporal Interest Points (STIP) [18], and Motion History Images (MHI) [19]. These features are typically combined with classifiers such as Support Vector Machines (SVMs) [20], K-Nearest Neighbors (KNN) [21], and Random Forests [22]. Unlike the traditional machine learning approaches, deep learning methods [23] have been demonstrated to provide a more robust, scalable, and effective solution by automatically learning rich representations from large datasets, allowing them to better address the variability and dynamics of aerial video data. Deep neural network architectures have unified both components in a seamless manner and has importantly enhanced the classification performance. Example of representative works include approaches relying on Convolutional Neural Networks (CNNs) [24], Graph Convolutional Networks (GCNs) [25], Recurrent Neural Networks (RNNs) and their variants such as Gated Recurrent Units (GRUs) and Long-Short Term Memory (LSTMs) [26], and Vision Transformers (ViTs) [27]. There has been a trend toward using pose information in the form of extracted keypoints in DNN frameworks [28], for human action recognition under the motivation that different action types are better distinguishable based on encoding bodily movements. Additionally, pose-based approaches are more robust to deal with background and illumination changes. Some pose-based approaches employed transformer-based networks to show promising results, however, they may not be directly deployable for action recognition in aerial settings due to view-point changes and motion dynamics (motion blur and jitters) caused by UAVs. Other methods work either with solo actions or interactions [29–31]. Furthermore, the need remains to devise lightweight deep models to aid ease and effectiveness in their deployment in real-world applications where computational cost and complexity are critical considerations.

To this end, this paper presents an effective lightweight Transformer model utilizing YOLO v8 pose estimator to extract keypoints for human action recognition in aerial videos. The proposed model (InterAcT) is generic in terms of being capable of recognizing both solo actions and interactions in a computationally efficient and effective manner. We have performed a thorough performance evaluation and comparison of the proposed method with several related state-of-the-art approaches on two well-known public datasets: Drone Action dataset [32] and UT-Interaction dataset [33], altogether comprising of a total of 18 classes including both solo actions and interactions. The proposed method outperforms the existing methods both in terms of accuracy and computational cost.

The contributions of this paper are as follows. First, a generic unified keypoints-based transformer model (InterAcT) is proposed, that is capable of recognizing both solo actions and human-human interactions in aerial videos. The second contribution is the optimization using exhaustive grid searches on various architectural parameters as part of training and validation in order to build an optimized and improved transformer-based framework, which is computationally efficient as well as accurate for real-world applications. The third contribution is the robustness and effectiveness of the proposed model, for which a detailed evaluation and comparison is conducted using multiple standard metrics to demonstrate the superior performance of the proposed method over several state-of-the-art methods on two well-known publicly available datasets. Lastly, we have made our model accessible online [34] to enable its use for recognition tasks and to support reproducibility of the reported results.

### Related work

While there exist several action recognition methods [11], only a limited number of approaches focused on addressing the problem for aerial videos [35]. Moreover, there are fewer methods that are generic to recognize both solo actions and human-human interactions.

Authors in [36] proposed a parts-based model with FCN that incorporates ORB attributes and texton maps for full body features, and Radon transform and 8-chain freeman codes for keypoints features to recognize solo action as well interaction classes. However, the model has limitations in terms of effectively dealing with recognition in aerial datasets due to low resolution and fast camera motion. In [37], the authors proposed a method that utilized extracted pose information with SVM and Random Forest to recognize violent actions in aerial videos; however, they demonstrated the effectiveness of their method on solo actions only. The authors in [38] presented a model that incorporates DWT, LBP and HOG to extract distinct features and used a multiclass SVM classifier with one-vs-one architecture to recognize action classes in aerial videos; however, their method is computationally costly due to the need to extract the conventional features and may not be suitable to be deployed in real-time applications. ST-PCANet [39] is a two-stream network that used unsupervised PCANet with features encoding schemes including BoF and VLAD followed by SVM classifier to classify actions in videos. The authors in [40] proposed an integrated X3D expanding architecture that utilized 3D ConvNets as a baseline model, flexible to handle occlusion and viewpoint changes in aerial videos and recognized solo actions only. The computational performance of the model may, however, not be desirable due to model complexity. The authors in [41] proposed a disjoint multi-task learning method that utilized a 3D CNN framework to recognize solo actions in aerial camera settings. They used GAN generated videos using videos synthetically created from GTA and FIFA games for human action recognition in real-world aerial videos when a few real aerial videos are available. However, generating representative GAN videos for all classes may not be feasible and games videos may have some bias toward some specific actions. The work on 3D Convolutional Neural Networks (3D CNNs) as presented in [42] is an extension of 2D CNNs that incorporated temporal dimension to capture temporal patterns of activity in videos. However, the computational cost of 3D CNNs models may be an issue due to a need of a large amount of annotated data to train. The work in [43] presented AR3D models to combine 3D CNN, residual structure, and attention mechanism to address above limitation. The method showed encouraging performance; however, the challenge pertaining to computational cost still remains. Graph Convolutional Networks (GCNs) [44] including 2P-GCN, Graph Diffusion Convolutional Network, AI-GCN, CTR-GCN and SGN and K-GCN were proposed that also relied on pose estimation but they generally aimed at human-human interactions only without accounting for solo actions. The authors in [45] presented an H-LSTM model that captured long-term inter-related dynamics among a group of persons for human interaction recognition. In [46], the keypoints-based LSTM model was proposed for recognizing solo actions in aerial camera settings. The work presented in [30] utilized keypoints-based transformer model that recognized solo actions in videos captured with fixed camera settings. Their work was extended in [31] that recognized solo actions in aerial videos.

Based on the review of related work, it is evident that there is need to devise a robust solution for human action recognition in aerial videos that is generic to work effectively both with solo actions as well interactions while being computationally efficient. Moreover, the transformer networks are being increasingly employed to solve several vision problems, but remains comparatively less explored for human action recognition task. Within transformer-based models, the keypoints-based transformer models have been relatively less investigated for their effectiveness, accuracy and performance in recognizing a large number of diverse action categories, both in fixed camera settings as well as aerial settings.

## Method

### Proposed transformer-based action recognition framework

**Fig 1:**
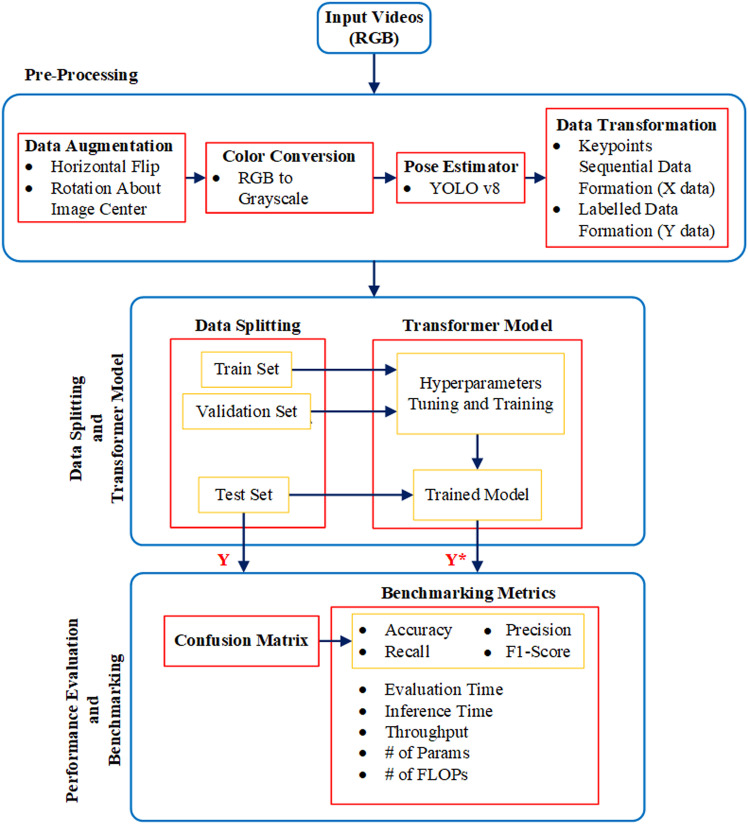
Flowchart illustrating different stages from input to output in the framework. Y represents true labels, while Y* represents predicted labels.

The proposed method utilizes body keypoints extracted using YOLO v8 pose estimation model [47]. The extracted keypoints data is preprocessed to transform data into the desired shape, which is then fed into the proposed Transformer framework for training and testing. The proposed model is inspired from “micro” Transformer architecture in earlier work [30] that was used for recognizing solo actions with ground-based videos. Unlike the “micro” Transformer architecture [30], the proposed framework offers lightweight optimized architectural settings to build a model that works effectively for both solo actions and interactions in a computationally efficient manner without compromising on performance accuracy. Fig 1 illustrates different stages in the framework from an implementation viewpoint.

#### Pose estimation.

Numerous pose estimation models including Open Pose [48], Alpha Pose [49], Hyper Pose [50], Blaze Pose [51], YOLO v7 [52] and YOLO v8 [47] exist to extract 2D or 3D keypoints. We used the widely used YOLO v8 that has reported high detection performance and low computational cost [31,47]. It extracts 17 keypoints per person in each frame of the videos. YOLOv8 pose model uses a backbone based on Cross Stage Partial Network (CSPNet) blocks and Path Aggregation Network (PANet). It enhances multi-scale detection and gradient flow, enabling efficient keypoint prediction. It is pretrained on extensive pose-annotated datasets like COCO and OpenPose due to which it accurately identifies poses across diverse activities. It divides the image into a grid and predicting the positions of key body parts within each grid cell, allowing it to process and analyze human poses in a single forward pass through the network.

**Fig 2:**
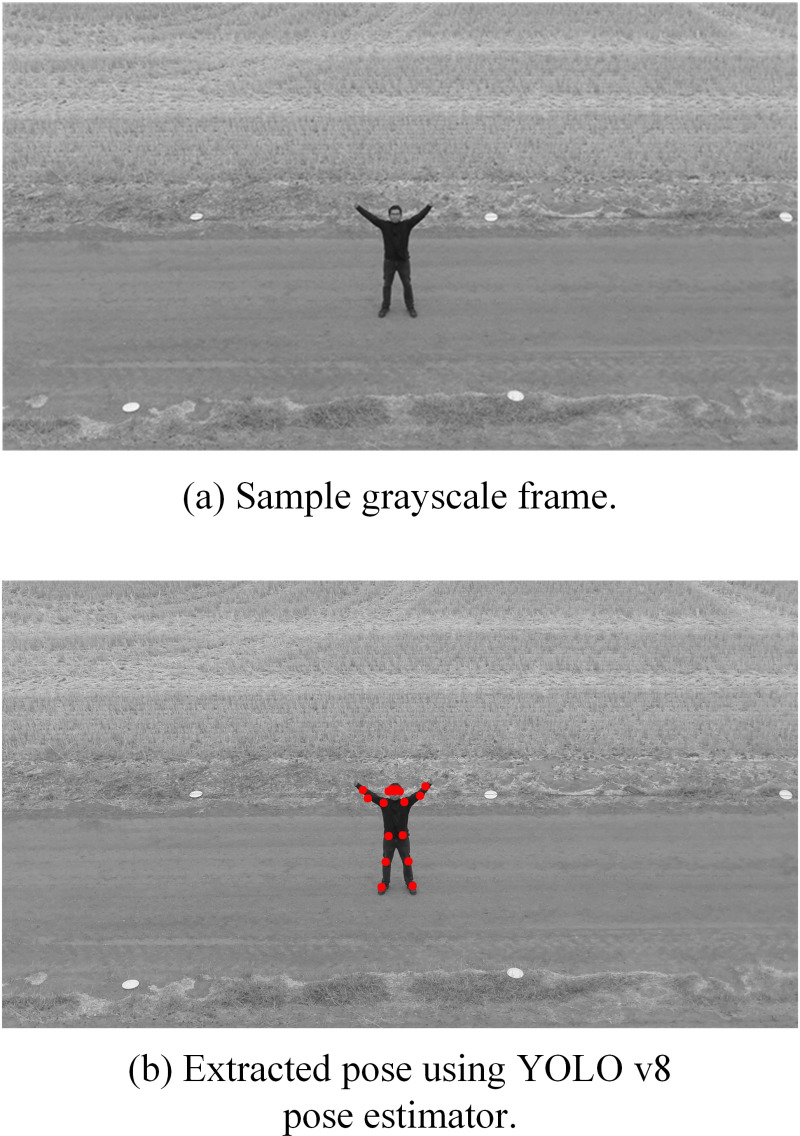
Sample image frame from the Drone Action dataset and its extracted keypoints using YOLOv8 pose model. Republished from [53] under a CC BY license, with permission from the copyright owner of the Drone Action dataset, original copyright [2019].

YOLO v8 pose model takes an input video of shape (*F,H,W,C*) where *F* is the total number frames, each frame having a dimensions of height (*H*) x width (*W*), and *C* denotes the number of input channels per frame. It returns output as (*F,K*), where *F* denotes the frame and *K* denotes the keypoints array. The extracted keypoints data is preprocessed into the desired shape to be fed into Transformer network for training and testing it. Fig 2 below illustrates the extracted keypoints for sample frames of “waving hands” solo-action class (top) and “handshaking” interaction-class (bottom). For keypoints extraction, we used grayscale imagery that has encouraging detection performance as well as a reduced computation cost.

#### Transformer-based action recognition model.

**Fig 3:**
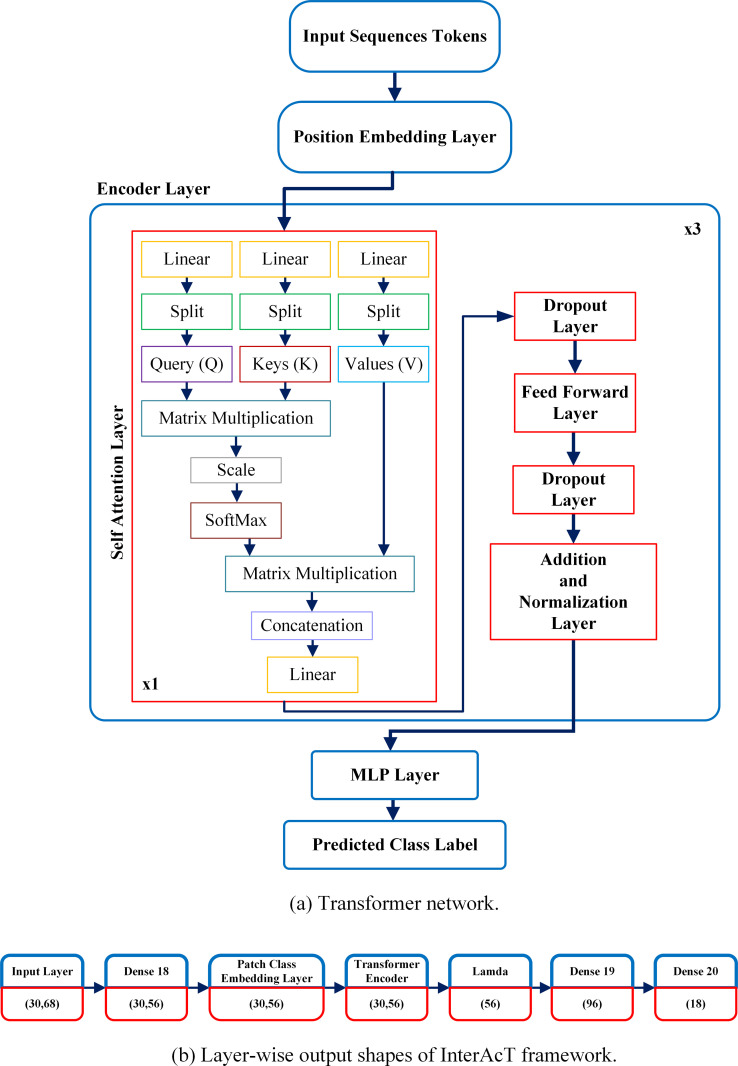
Overview of InterAcT architecture. (a) Image illustrating modules of the transformer network; (b) image illustrating the output shape of different layers.

Fig 3 illustrates the proposed model architecture. The backbone of the framework is the Transformer encoder layer, which comprises of multiple layers employing a self-attention layer and feed-forward blocks. After each block, the dropout, layer-normalization, and residual connections are applied. Each feed-forward block operates as a multi-layer perceptron using GeLu non-linearization. The memory block that learns the temporal pattern of the input sequences in the Transformer network is the self-attention block. The input sequences tokens are fed into it as linearly transformed vectors known as Query (*Q*), Key (*K*) and Values (*V*). Using *Q* and *V* vectors, the attention weights matrix is computed, which further multiplied with *V* vector gives the self-attention output as per Equation 1. At the final stage, class tokens are fed into the MLP head that output a logit vector, on which softmax is applied to predict the class label.

The preprocessed keypoints for each class comprises of $S_{N}$ sequences where *N* denotes the number of sequences per class. Each sequence has a shape (*F,K*), where *F* denotes the number of frames per sequence which is set to 30 in our case and *K* denotes the number of keypoints computed as follows: $P_{N}$*$K_{N}$*$C_{N}$, where $P_{N}$ is maximum required number of persons in the videos, $K_{N}$ denotes the number of keypoints extracted per person and $C_{N}$ is the number of coordinates/channels per keypoint. In our case, *K* is set to 68. For each sequence, the Transformer network will extract temporal features using 30 frames in it. First, the keypoints are transformed linearly into an Embedding matrix which are added with Positional Embedding matrix that has learnable parameters provides the positional information of each frame thus forms an embedded matrix $X_{emb}$. The embedded matrix $X_{emb}$ has shape of (*F,* $d_{model}$), where $d_{model}$ denotes the embedded dimension of each vector (row) of $X_{emb}$. The Query (*Q*), Key (*K*) and Values (*V*) vectors are generated using the $X_{emb}$ as given by Equations 2–4 respectively.


$$Self\;Attention\;=\;Softmax\left(\frac{QK^{T}}{\sqrt{d_{K}}}\right)V$$ (1)

$$Q\;=\;X_{emb}W_{Q}$$ (2)

$$K\;=\;X_{emb}W_{K}$$ (3)

$$V\;=\;X_{emb}W_{V}$$ (4)

Where $W_{Q}$, $W_{K}$ and $W_{V}$ are the weights matrices having learnable parameters and their dimensions are kept same in the network, i.e., $d_{Q}=d_{K}=d_{V}$, which makes the dimensions of *Q*, *K* and *V* the same, i.e., (*F*, $d_{model}=d_{Q}=d_{K}=d_{V}$). In our case $d_{model}$ is set to 56. The self-attention output weight matrix is transformed by a layer having a weight matrix $W_{0}$ which has the shape ($d_{V}$, $d_{model}$). As $d_{model}=d_{Q}=d_{K}=d_{V}$, so the shape of $W_{0}$ becomes ($d_{model}$, $d_{model}$). This transformation makes the output shape of self-attention block equal to (*F*, $d_{model}$). This output matrix is then fed into the feed forward network that linearly transforms the output using the operations as given by Equation 5.


$$Feed\;Forward\;(x)\;=\;max\left(0,\left(xW_{1}+b_{1}\right)\right)W_{2}+b_{2}$$ (5)

Where *x* is the output of the attention block. $W_{1}$ and $W_{2}$ are the weights matrices having shapes ($d_{model},d_{FF}$) and ($d_{FF},d_{model}$) respectively. $b_{1}$ and $b_{2}$ are biases vectors, both having the same shapes (*F*,). In our case, $d_{FF}$ = $d_{model}$. The output of feed forward is then fed into the MLP head to predict the class label. The layer-wise output shapes of InterAcT model are illustrated in Fig 3b.

## Experimental setup

This section presents the experimentation details including the description of the datasets and the system specifications that are used in training and testing the model.

### Datasets

We used two publicly available datasets for evaluation, the Drone Action dataset [32,53] and UT-Interaction dataset [33,54]. Indeed, the choice of the two datasets is made taking into account the presence of solo actions and human-human interactions, which this study focuses on.

**Fig 4:**
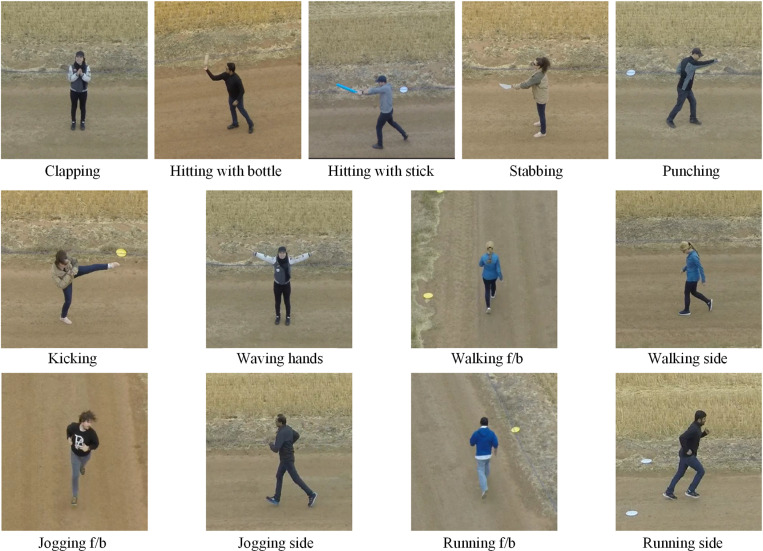
Sample image frames of solo action classes from the Drone Action dataset. Republished from [53] under a CC BY license, with permission from the copyright owner of the Drone Action dataset, original copyright [2019].

We have used 13 solo actions classes from the Drone Action dataset [32,53] as illustrated in Fig 4. It contains RGB videos having spatial resolution of 1920x1080, recorded at a frame rate of 25 fps in an outdoor environment with a low-altitude and slow-speed moving drone. The solo actions are performed by 10 actors on an unsettled road near wheat fields. It contains challenges of cluttered background and viewpoints changes.

Also, we have used 5 human-human interaction classes from the UT-Interaction dataset [33,54], with corresponding illustrations available online [34]. It contains RGB videos having spatial resolution of 720x480, recorded at a frame rate of 30 fps in an outdoor environment with a low-altitude camera. The human-human interactions are performed by 6 actors in two different scenarios: a parking lot and a lawn on a windy day. It contains challenges of camera jitters, varying zoom rates, illumination changes and cluttered backgrounds. Table 1 gives the summary of both datasets. Our study aims on recognizing a wide range of actions and interactions captured by low-altitude flying drones, making these two datasets highly suitable and relevant for our research.

**Table 1:** Summary of the datasets.

Dataset	Year Published	Action Type	Modality	Frame Rate	Resolution	Viewpoint	Number of Actors	Number of Classes
Drone Action [32,53]	2019	Solo Actions	RGB	25 fps	1920x1080	Aerial	10	13
UT-Interaction [33,54]	2010	Human-Human Interactions	RGB	30 fps	720x480	Aerial	6	5

Both datasets contain varying numbers of videos per class, leading to an imbalance in the amount of extracted sequential keypoints data. This class imbalance can affect model performance, potentially limiting its accuracy and effectiveness. Data augmentation techniques are employed to address this issue, particularly for classes with fewer videos, allowing the extraction of sufficient sequential data for model training and improving model generalizability. To increase the number of sequential samples, it’s crucial to augment the dataset with additional videos. Numerous augmentation techniques can be utilized for this purpose. In our case, we employed two methods that are horizontal flipping and rotation – both effectively simulate realistic yet diverse drone perspectives enabling the evaluation of robustness of the proposed model to variations in aerial settings. These techniques help increase both the number of videos and the corresponding sequential data per class. Despite the formation of sequential data, class imbalance persists. To address this, data slicing is employed, where the class with the fewest sequences is used as a reference. All classes are then sliced to match this number, ensuring balanced sequential data across classes. The class-wise data statistics are given in Table 2. In this study, the preprocessed keypoints data (balanced) is split into three sets. For training and validation, we used 80% training set and 10% validation set, respectively. For performance evaluation, we used a 10% test set.

**Table 2:** Class-wise statistics.

Dataset	Class Name	Number of Videos	Number of Extracted Keypoints Frames	Number of Sequences	
**Fixed Window**	**Sliding Window**
**Drone Action** [32,53]	Clapping	10	2794	93	2765
	Hitting bottle	20	6294	210	6265
	Hitting stick	20	6380	213	6351
	Jogging fb	40	3354	112	3325
	Jogging side	20	3738	125	3709
	Kicking	20	6849	228	6820
	Punching	20	5100	170	5071
	Running fb	80	2678	89	2649
	Running side	40	4889	163	4860
	Stabbing	20	6367	212	6338
	Walking fb	20	3336	111	3307
	Walking side	20	5206	174	5177
	Waving hands	10	3565	119	3536
**UT-Interaction** [33,54]	Hand shaking	40	3878	129	3849
	Hugging	40	2751	92	2722
	Kicking	40	1754	58	1725
	Punching	80	3436	115	3407
	Pushing	40	2085	70	2056
**TOTAL (imbalanced)**		580	74454	2483	73932
**TOTAL (balanced)**			31572	1044	31050

### System specifications

This section presents the hardware and software resources that are utilized to perform the experimental evaluation of the proposed framework. Table 3 lists the specifications of the hardware and Table 4 shows the software requirements.

**Table 3:** Hardware specifications.

Component	Specifications
Processor Name	12^th^ Generation Intel (R) Core (TM) i7-12700
Processor Clock Speed	4.9 GHz
Processor Cores	12
Processor Threads	24 (2 Threads per Core)
Graphics Card Name	NVIDIA GeForce RTX 3090
Graphics Memory (VRAM)	24 GB
RAM Size	62 GB
RAM Technology	DDR5

**Table 4:** Software specifications.

Component	Specifications
Programming Language	Python
Python Version	3.11.5
Deep Learning Framework	TensorFlow
TensorFlow Version	2.15.0
Operating System	Linux (Ubuntu 22.04.1)
Linux Kernel Version	6.5.0-25-generic

## Results and discussion

This section presents the experimental results of our model in a systematic manner. Specifically, it describes model parameters tuning and training, followed by evaluation and comparison of the proposed model with existing related models.

### Model parameters tuning

We performed an exhaustive grid search on architectural parameters as well as training hyperparameters to obtain a light-weight architecture with optimized parameter settings. Tables 5 and 6 shows the architectural parameters and the training hyperparameters that are experimented as part of this study. The architectural parameter tuning is performed sequentially with following initial training settings: fixed window sequential data, 500 training epochs, sequence length of 30, AdamW optimizer with learning rate of 0.0001 and weight decay of 0.00001, GeLU activation function and a batch-size of 32. The optimized values of architectural parameters/hyperparameters are given in Tables 5 and 6, respectively.

**Table 5:** Architectural parameters of the proposed model.

Parameters	Set of Values	Optimized Value
Number of Self-Attention Heads (H)	1	1
Number of Transformer Encoder Layers (L)	1, 2, 3, 4	3
Embedded Dimensions ($D_{model})$	1, 2, 4, 8, 12, 16, 24, 32, 40, 48, 56, 64	56
Dropout Percentage	0.05, 0.1, 0.15. 0.20, 0.25, 0.30	0.10
Dimensions of MLP Head ($D_{mlp}$)	1, 2, 4, 8, 16, 32, 64, 96, 128, 160, 192, 224, 256	96

**Table 6:** Hyperparameters of the proposed model.

Hyperparameters	Set of Values	Optimized Value
Epochs	500	500
Sequence Length	30	30
Optimizers	AdamW, LAMB, LazyAdam, RAdam, SGDW	AdamW
Learning Rates	0.1, 0.01,0.001,0.0001,0.00001,0.000001	0.001
Weight Decays	0.1, 0.01,0.001,0.0001,0.00001,0.000001	0.000001
Activation Functions	SeLU, SiLU, ELU, GeLU, Swish, Mish	GeLU
Batch sizes	1,2,4,8,16,32,64,96,128,160,192,224,256	192
Sequential Data Formation – Types	Fixed Window, Sliding Window	Sliding Window

**Fig 5:**
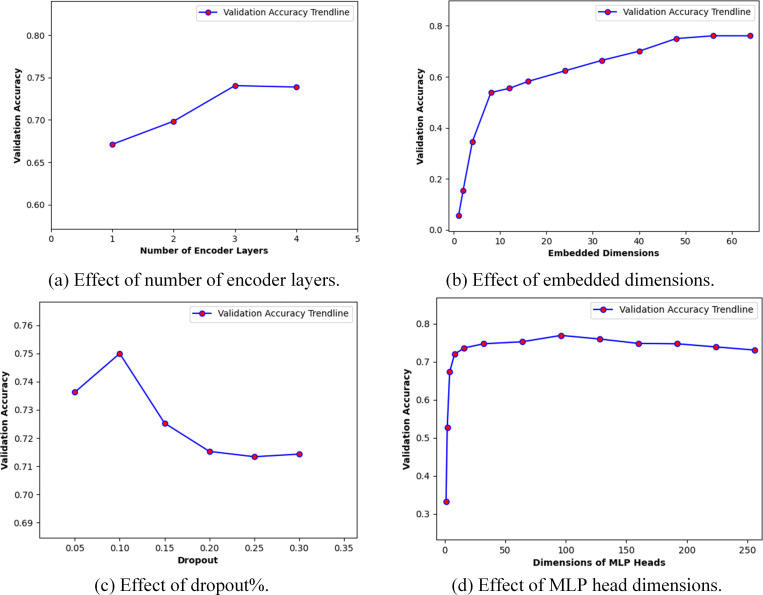
Effect of variation of architectural parameters.

The results of architecture parameter tuning are shown in Fig 5. Fig 5a illustrates the effect of varying the number of encoder layers; it shows that the validation accuracy increases with the increase in the number of encoder layers and the performance tends to stabilize at 3 encoder layers giving the highest validation accuracy of 0.7404. Fig 5b illustrates the effect of embedded dimensions; it indicates an increasing validation accuracy with increase in embedded dimensions and the highest validation accuracy of 0.7610 is obtained at embedded dimensions of 56. Fig 5c illustrates the effect of varying the dropout percentage parameter; the validation accuracy decreases with an increase in the dropout percentage because most of the input units are set to zero during training, thus leading the model to under-fitting. The highest validation accuracy of 0.7500 is obtained at a dropout of 0.10. Fig 5d illustrates the effect of varying the MLP head dimensions; a highest validation accuracy of 0.7692 is obtained at MLP head dimensions of 96. Thus, the selected architectural parameters for our model are: three encoder layers, embedding dimensions of 56, a dropout rate of 0.10, and MLP head dimensions of 96.

**Fig 6:**
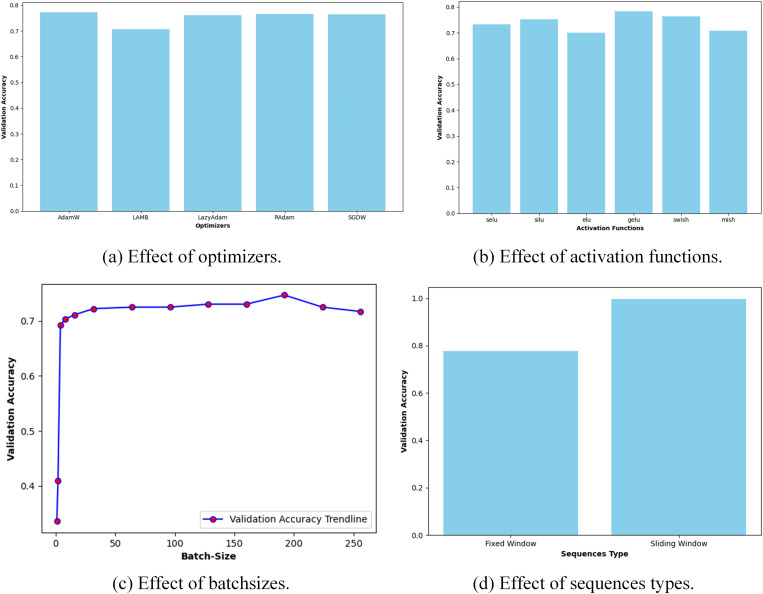
Effect of variation of hyperparameters.

Similarly, the results of hyperparameters tuning are illustrated in Fig 6. The result of optimizers with different learning rates and weight decays is illustrated in Fig 6a. Among the optimizers, AdamW with learning rate of 0.001 and weight decay of 0.000001 provides the highest validation accuracy of 0.7720. Fig 6b shows the effect of activation functions. Among the activation functions, GeLU gives the highest validation accuracy of 0.7830. Fig 6c illustrates the effect of batch sizes; it shows that increasing batch size increases validation accuracy. Increasing batch size leads to a fast convergence in training and reduces training time. However, increasing it significantly could lead to reduced performance. A highest validation accuracy of 0.7473 is obtained at a batch size of 192. Fig 6d illustrates the comparison between sequences with a fixed window or sliding window for training the model. It is clear that the sliding window reports a higher validation accuracy of 0.9967 due to the increased number of sequences. On the other hand, the fixed window results in fewer sequences for model training; hence, the model performance drops as compared to sliding window. Thus, the selected training parameters for our model are: AdamW optimizer with learning rate of 0.001 and weight decay of 0.000001, GeLU activation function, a batch size of 192 and sliding window sequences.

### Model training

**Fig 7:**
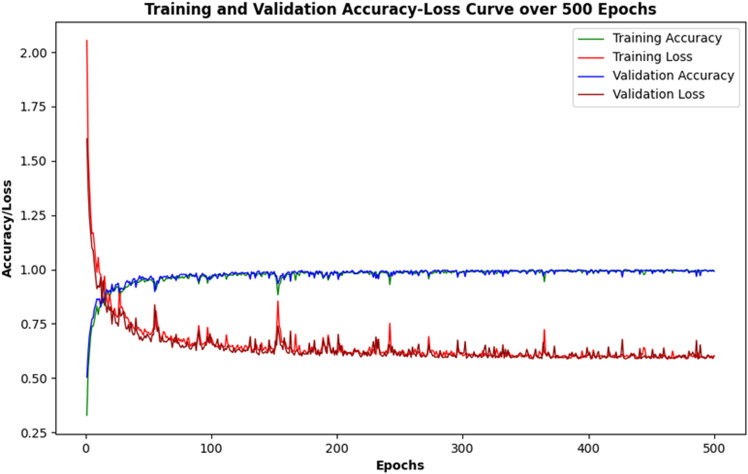
Training and validation accuracy/loss curves.

After tuning parameters of the model, we trained and evaluated it. The training and validation accuracy-loss curves are shown in Fig 7. From Fig 7, both training and validation accuracies are increasing, and losses are decreasing over each epoch, thus showing that the model is gradually learning. We trained the model for 500 epochs.

### Performance evaluation and comparison

This section presents the performance evaluation and comparison of our model with the existing AcT models and other state-of-the-art approaches to demonstrate its effectiveness and robustness. Table 7 below shows the architectural-level comparison of our model with the existing AcT models [30]. From this table, it is clear that our model has the optimized settings resulting in a light-weight architecture.

**Table 7:** Architectural comparison with AcT models [30].

Model Architecture		Multi-head Attention Layers	Encoder Layers	Embedded Dimensions	Drop-out Percentage	MLP Heads Dimensions
**AcT** [30]	Micro	1	4	64	0.30	256
	Small	2	5	128	0.30	256
	Base	3	6	192	0.30	256
	Large	4	6	256	0.40	512
**Ours**		**1**	**3**	**56**	**0.10**	**96**

**Fig 8:**
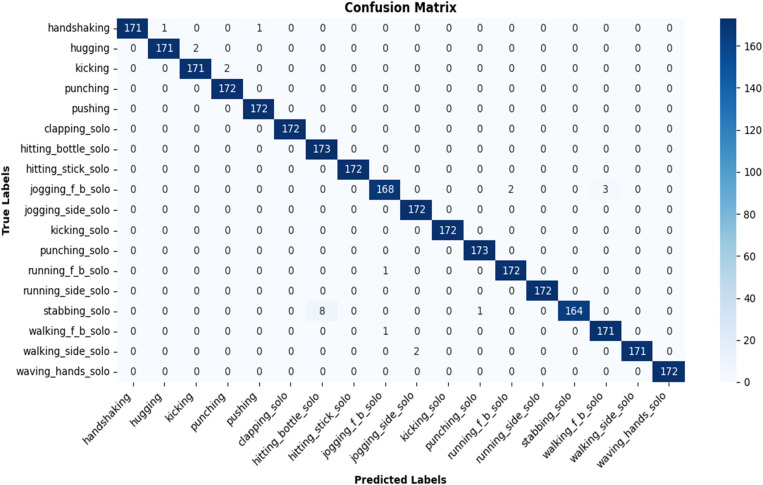
Confusion matrix for 18 classes comprising both solo actions and human-human interactions.

**Fig 9:**
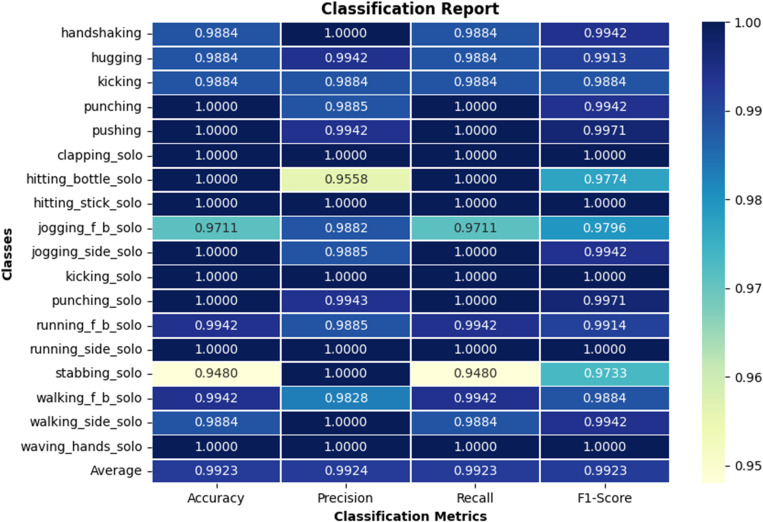
Classification scores for 18 classes comprising both solo actions and human-human interactions.

**Fig 10:**
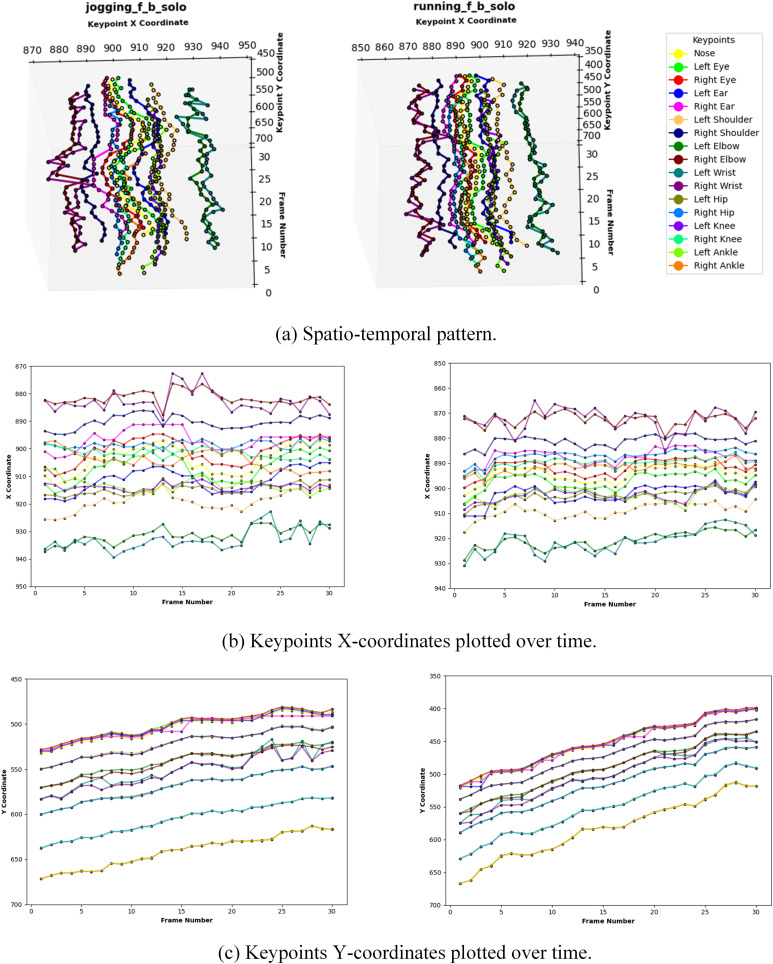
Column 1: a jogging_f_b_solo case that is correctly predicted as jogging_f_b_solo. Column 2: a running_f_b_solo case that is misclassified as jogging_f_b_solo due to similar spatio-temporal trends.

For performance evaluation, the computed confusion matrix and the classification report including class-wise accuracy, precision, recall and F1-scores, are illustrated in Figs 8 and 9, respectively. It is evident that the performance is generally very encouraging for all classes with some exceptions where there are misclassifications due to similar spatio-temporal trends (Fig. 8). For example, Fig 10 illustrates a deeper insight into an instance where *jogging_f_b_solo* is misclassified as *running_f_b_solo*. This is because both actions exhibit similar spatio-temporal pattern, making it difficult for the model to distinguish between them.

We also compared the performance of our model with several traditional deep learning models including 2P-GCN [55], LSTM [46], 3D-ResNet [56] and 3D-CNN [57] as well as four state-of-the-art AcT models [30] as shown in Table 8. Table 8 show multiple performance metrics: *Model parameters* refer to the total learnable weights in the model, *model FLOPs* (floating point operations) indicate computational complexity, *evaluation time* is the duration for assessing model performance on the test set, *inference time* is the time taken to make a single prediction, *throughput* measures the number of sequences processed per second, *accuracy* reflects the percentage of correct predictions, *precision* is the proportion of correctly predicted positive instances among all positive predictions, *recall (sensitivity)* is the proportion of correctly predicted positive instances among all actual positives, and *F1 score*, the harmonic mean of precision and recall, provides a single measure that balances both precision and recall for assessing model performance in case of class imbalance. From Table 8, it is clear that our model outperforms all the models on multiple performance metrics. These findings show a better suitability of our model for deployment in real-time applications as compared to the other models.

**Table 8:** Performance comparison with state-of-the-art methods.

Model			Model Parameters (M)	Model Flops (G)	Evaluation Time (s)	Inference Time (ms)	Throughput (Sequences/s)	Accuracy	Precision	Recall	F1-Score
**GCN**	2PGCN [55]		4.0300	2.5100	8.900	2.87	349	0.9337	0.9438	0.9337	0.9328
**RNN**	LSTM [46]		4.1566	0.1644	7.6194	2.45	408	0.9774	0.9774	0.9773	0.9773
**CNN**	3D ResNet [56]		33.1567	0.3241	1.2067	0.389	2573	0.9921	0.9921	0.9921	0.9921
	3D CNN [57]		0.3524	0.0112	0.9509	0.306	3265	0.9920	0.9923	0.9920	0.9920
**Transformer**	**AcT** [30]	Micro	0.2277	1.0520	0.9353	0.301	3320	0.9353	0.9354	0.9353	0.9352
		Small	1.0419	1.4881	0.9893	0.312	3139	0.9893	0.9897	0.9893	0.9894
		Base	2.7426	2.1741	0.9907	0.319	3134	0.9907	0.9908	0.9907	0.9907
		Large	4.9052	3.0388	0.9558	0.308	3249	0.9558	0.9558	0.9558	0.9558
	**Ours**		**0.0709**	**0.0389**	**0.2013**	**0.0648**	**15425**	**0.9923**	**0.9924**	**0.9923**	**0.9923**

## Conclusions and future work

In this paper, we presented an efficient and effective generic keypoints-based transformer model, called InterAcT, which is capable of recognizing solo actions as well as human-human interactions in aerial videos, by utilizing the bodily keypoints extracted using YOLO v8 pose estimator. Our optimized model, comprising 0.0709 million parameters and 0.0389 Gflops, has demonstrated encouraging performance on the UT-Interaction and Drone-Action datasets. With sliding sequential data, the model achieves a high accuracy of 0.9923, outperforming the AcT models (micro: 0.9353, small: 0.9893, base: 0.9907, and large: 0.9558), 2P-GCN (0.9337), LSTM (0.9774), 3D-ResNet (0.9921), and 3D CNN (0.9920). Moreover, our model also shows better performance than other models in terms of evaluation time, inference time, throughput, precision, recall, F1-score, as well as in terms of model parameters and model flops. The model’s performance combined with its lower computational complexity, highlights its efficiency and robustness in comparison to several existing models. The key strength of the model lies in its lightweight architecture, thus making it more deployable in several real-world applications such as aerial surveillance, public safety monitoring systems, private security monitoring systems, home surveillance, healthcare systems, retail and customer monitoring systems and autonomous vehicle systems.

As future work, the proposed model can be adapted to incorporate other action categories such as body gestures and multi-person human-human interactions. Moreover, it would be interesting to explore the use of multi-modal data by fusing sensors (non-visual) data with visual data, in an attempt to further enhance recognition performance in extreme weather and environmental conditions. Furthermore, it would be useful to deploy and perform evaluation of the proposed framework on resource constrained devices to further test the effectiveness.
